# Mesoscale movement and recursion behaviors of Namibian black rhinos

**DOI:** 10.1186/s40462-019-0176-2

**Published:** 2019-11-09

**Authors:** Dana Paige Seidel, Wayne L. Linklater, Werner Kilian, Pierre du Preez, Wayne M. Getz

**Affiliations:** 1 Department of Environmental Science, Policy, and Management, University of California, Berkeley, Mulford Hall, Berkeley, CA, USA; 2 Centre for Biodiversity and Restoration Ecology, Victoria University of Wellington, P.O. Box 600, Wellington, 6140 New Zealand; 3 Centre for African Conservation Ecology, Nelson Mandela University, Port Elizabeth, South Africa; 4 Etosha National Park, PO Box 6, Okaukuejo via Outjo, Namibia; 5 School of Mathematical Sciences, University of KwaZulu-Natal, Durban, South Africa

**Keywords:** *Diceros bicornis*, Black rhino, Animal movement, Recursion, Displacement, Home range, T-LoCoH, NDVI

## Abstract

**Background:**

Understanding rhino movement behavior, especially their recursive movements, holds significant promise for enhancing rhino conservation efforts, and protecting their habitats and the biodiversity they support. Here we investigate the daily, biweekly, and seasonal recursion behavior of rhinos, to aid conservation applications and increase our foundational knowledge about these important ecosystem engineers.

**Methods:**

Using relocation data from 59 rhinos across northern Namibia and 8 years of sampling efforts, we investigated patterns in 24-h displacement at dawn, dusk, midday, and midnight to examine movement behaviors at an intermediate scale and across daily behavioral modes of foraging and resting. To understand recursion patterns across animals’ short and long-term ranges, we built T-LoCoH time use grids to estimate recursive movement by each individual. Comparing these grids to contemporaneous MODIS imagery, we investigated productivity’s influence on short-term space use and recursion. Finally, we investigated patterns of recursion within a year’s home range, measuring the time to return to the most intensively used patches.

**Results:**

Twenty four-hour displacements at dawn were frequently smaller than 24-h displacements at dusk or at midday and midnight resting periods. Recursion analyses demonstrated that short-term recursion was most common in areas of median rather than maximum NDVI values. Investigated across a full year, recursion analysis showed rhinos most frequently returned to areas within 8–21 days, though visits were also seen separated by months likely suggesting seasonality in range use.

**Conclusions:**

Our results indicate that rhinos may frequently stay within the same area of their home ranges for days at a time, and possibly return to the same general area days in a row especially during morning foraging bouts. Recursion across larger time scales is also evident, and likely a contributing mechanism for maintaining open landscapes and browsing lawns of the savanna.

## Background

Movement ecology holds considerable promise for understanding rhino ecology and their conservation. However, an ISI Web of Science keyword search using “movement ecology” and “rhinoceros” yields no published studies. Make the same search for elephant or whales and many published studies appear. It is interesting to contemplate why rhino are so much less studied in this regard. Foremost among the reasons is probably that elephant and whales range over great distances and migrate, thus making movement a more obvious feature of their ecology and germane to their conservation and management. Rhinos, however, are attached to comparatively small home ranges with intensively used core areas [1]. Movement ecology studies of other large herbivores that are similarly sedentary, such as giraffe and hippopotamus, are also scant. Fitting GPS collars to rhino has also proved more difficult [2] and controversial [3, 4] than many other species, including elephants. Rhinos do not have slender necks to hold a collar between their heads and shoulders; and they treat their collars roughly, breaking them on the vegetation and rocks on which they push and rub, or reducing satellite antennae functionality through a coating of mud when wallowing. Until recently, rhino movement studies were limited to short-range horn-implant transmitters [5, 6] that yielded comparatively small amounts of discontinuous movement data. These studies, however, provided movement data at both the micro (step-by-step foraging) and macro (seasonal ranging) scales, but not at the meso (daily or weekly) scale.

The study of movement is central to addressing the challenges of rhino species conservation because recovery now depends on growing and managing meta-populations in, largely fenced, wildlife reserves that are networked by rhino translocations for reintroduction and restocking. For example, simulating source-sink dynamics in the larger reserves has met with some, but mixed, success for rhino [7]. Evaluating its usefulness and limitations depends on our understanding of rhino movement, particularly dispersal [8]. Where an entire population is a donor for the meta-population, rapid compensatory reproduction depends also on dispersal and range recolonization [8]. Moreover, mitigating the significant environmental and social risks inherent in rhinos’ release into unfamiliar habitat and populations [6] depends also on anticipating the movement behavior of released individuals. Concern exists that competition with elephant [9] and calf depredation by large predators, such as spotted hyena and lion [10] might slow species recovery. Rhinos’ movements in relation to competitors and predators could be revealing and may address such concerns. Studies of home range have been crude and analyses are still plagued by facile comparisons [11, 12] that would be greatly improved by meso-scale movement analyses. Lastly, and perhaps most importantly, rhino habitat is increasingly shared with people and their infrastructure. They may be in rhinos’ habitat to view or hunt them or other wildlife, or they may be poachers. Rhino poaching continues to be the largest cause of population decline and places the greatest limits on rhino species recovery [13]. Movement of individual rhinos in response to encounters with humans, whether they be tourists or hunters, sanctioned or illegal, promises to facilitate co-existence and persistence in rhino habitats that are increasingly anthropogenic.

Movement has been particularly central to studies of rhino food and habitat choice, and the configuration of home ranges and territories. At the micro scale, feeding tracks have been the mainstay of monitoring and research for several decades, most often to understand food preferences [14–16]. Black rhinoceros are highly selective foragers. While shown to feed on a great diversity of plant species, both within and between reserves, they depend on fewer than 10 species for the majority of their diet in most places (e.g., [16, 17]). Habitat choices are similarly particular such that patterns of site-use are easily discernible everywhere that they have been measured (e.g., [18]). More than other large mammals, rhinos’ micro-scale movements have been studied because of the ease with which individuals could be identified and tracked over sandy or muddy substrates that constitute the landscape of many rhino populations [19, 20]. At the macro-scale, the description of home ranges or territories, especially using horn-implant transmitters, has also been common and applied to understand habitat requirements [12, 21–23]. Black rhino are a ubiquitously solitary living species [24] and quite unlike white rhinoceros in this regard. Groups, when they do form, are small and ephemeral, lasting a few days at a time. The longest inter-individual associations are mothers and calves and then mates (i.e., mate guarding that occurs over several days and even a few weeks). Adult, same-sex associations are uncommon and ephemeral. Greater male, than female, displacement is expected as male ranges are ordinarily regarded as territories and a successful bull’s territory typically overlaps more than one breeding females territory [24]. Nonetheless, missing from the literature on rhino is an understanding of movement at the meso-scale and thereby constricting our ability to answer the questions: How do micro-movements translate into home ranges and spatial-use patterns? Are rhino spatial and temporal movements cyclic? Can we use rhino movement to understand ecological processes other than foraging? Research on black rhino is yet to take best advantage of the tools and ideas of the (relatively) young sub-discipline of movement ecology.

Despite the need and opportunity, the application of concepts and tools from animal movement research to understanding rhino ecology and conservation has not advanced appreciably since the literature was last reviewed over 15 years ago [25]. Our understanding of the fundamental ecology of rhino movement is still rudimentary in that it relies largely on anecdotal observations at the micro- and macro-scales, rather than extensive sets of movement data. For example, it is routinely assumed that rhinos’ movements are driven by resource heterogeneity and optimal foraging of resource patches. We might expect, therefore, meso-scale movements to be bimodal, whereby short distance movements within patches are punctuated by fewer longer-distance movements between patches, especially in more arid environments. It is also assumed that daily drinking at a few sources of standing water, mostly soon after dusk or before dawn [14], causes rhino movements to be circuitous around waterholes. It is also known that rhino are crepuscular and thus assumed that the movement cycle is during active, mostly feeding periods around dawn and dusk [26, 27]. What is not known is whether daytime or nighttime feeding cycles generate more displacement and to what degree resting sites, such as waterholes, create centrality in meso-movement patterns.

Furthermore, rhinoceros, like other mega-herbivores, are considered to be ecological engineers [24] based on their potential to impose spatial and temporal heterogeneity on landscape vegetation structure and regulate other causes of disturbance, such as fire. For example, evidence suggests that white rhino grazing imposes vegetation heterogeneity on the landscape by creating grazing lawns. Those lawns appear to facilitate feeding by other smaller-bodied grazers and regulate fire extent and intensity [28]. A similar effect has been suggested for large browsers [29–31], such as black rhinoceros. Hedging of its favoured browse may make trees and shrubs more productive and prevent their growth into inaccessible height classes and vegetation succession towards a canopy. Thus, rhino movements should betray recursive movements, returning individuals repetitively to the same feeding areas with elevated productivity and biodiversity. Recursion may be critical to understanding rhino impact on habitat structure and ecosystem function, however, to our knowledge, this has not yet been rigorously studied using GPS relocation data from continuously monitored individuals.

In this manuscript, using a unique relocation dataset for 59 black rhinos (*Diceros bicornis*) ranging across national parks and community conservation parcels in northern Namibia, we seek to answer some of these questions, evaluate long-held assumptions, and build an empirical understanding of rhino recursion across scales. To begin, we explore 24-h displacement cycles at times of day associated with foraging and resting to understand daily movement behaviors and inspect tracks for evidence of circuitous movement. Secondly, we identify areas across rhinos’ ranges with high recursion or long-duration visits and explore how spatial-distribution of resources (i.e. functional water and productivity) may influence rhino movements and subsequent home range development. By exploring meso-scale movement in rhinos, our goal is contribute new foundational knowledge of rhino ecology and to identify useful next questions for future research of this important and threatened species and the biodiversity they both represent and support.

## Methods

### Telemetry data and study area

**Fig. 1:**
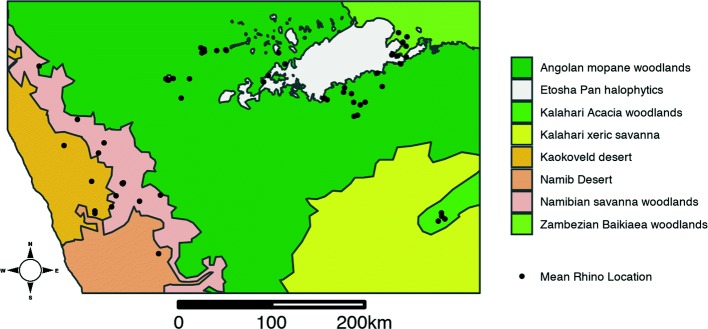
A map of the study region showing the distribution of geo-tagged individuals across Namibian ecoregions as specified by Dinerstein et al. [32]

The rhinos in this study had ranges in northern Namibia: in the unfenced Kunene Region to the west, in Etosha National Park to the east and in Waterberg National Park to the southeast. The ranges occur in several distinct ecoregions and across a marked precipitation gradient with the western coast being significantly dryer than the eastern region (Fig. 1; [32]). GPS locations were acquired from multiple studies across two sampling periods and multiple rhino clusters. All rhinos were fitted with IR-SAT Iridium satellite foot bracelets with GPS and UHF (AWT Wildlife Tracking, Pretoria, South Africa). Rhino immobilization was done from a helicopter by veterinarians from the Ministry of Environment and Tourism, Namibia. The capture, collaring, and transportation (when necessary) of rhinos was done following Standard Operating Procedures in compliance with the best veterinary practices.

**Fig. 2:**
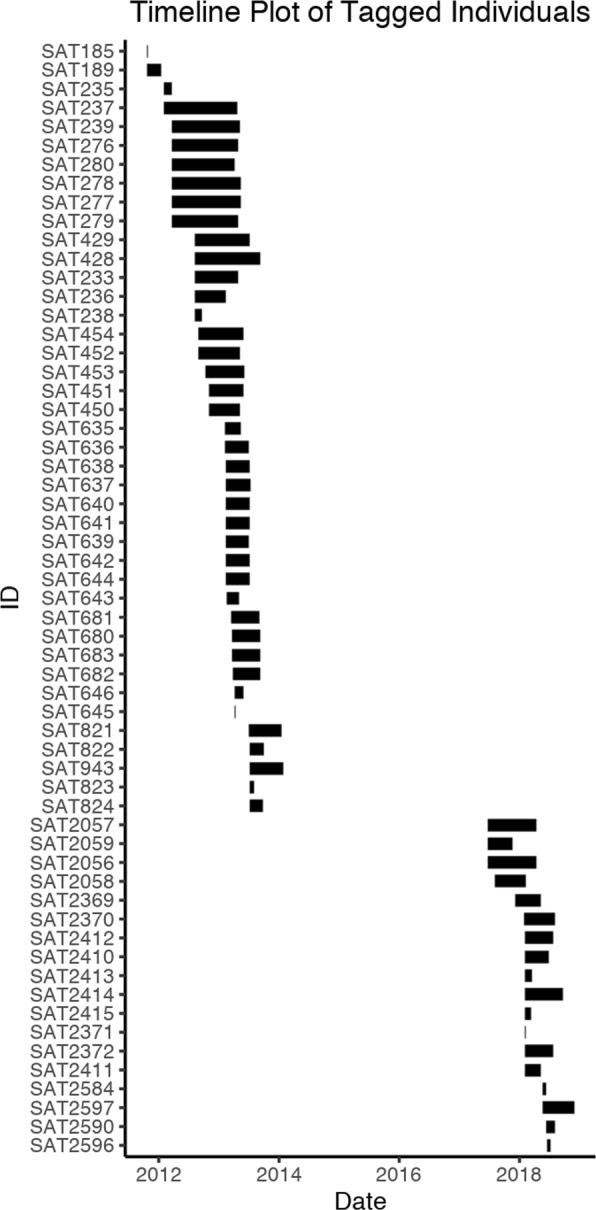
A timeline plot indicating sampling intervals for all individuals used in the study. Produced using R package stmove [33]

Our dataset includes relocations from 59 individual rhinos: 41 individuals were sampled between October 2011 and January 2014 and an additional 18 individuals were sampled between July 2017 and November 2018 (Fig. 2). Four rhinos ranged within Waterberg National Park, all of which were sampled in 2013. Sixteen individuals ranged freely to the west and south of Etosha National Park boundaries in community-based conservation lands. Finally, relocation data from across the full expanse of Etosha National Park was collected from 39 individuals. Thirty-eight of the rhinos were female, with 15 of those identified as pregnant or accompanied by a calf at capture. 20 rhinos were male and the sex of one individual was not recorded.

As is common among rhino telemetry studies, fix rates across all individuals were irregular ranging from roughly 1 fix per hour for some individuals to less than 3 fixes per day for other individuals. All trajectories had some degree of fix lag, missed fixes, or inconsistent fix rates whereby regularization would result in observation loss and interpolation methods would likely have biased movement analyses. Therefore, analyses were chosen and results interpreted with data irregularity in mind. Coordinates for 63 known waterholes across Etosha National Park were used in analyses to evaluate hypothesized recursion behaviors. All data manipulation and analyses were done in program R (v. 3.5.1) [34].

### Twenty four-hour displacement cycles

Rhinos are known to be crepuscular creatures, with their most active feeding periods around dawn and dusk and their longest period of rest during the heat of midday. To investigate whether daytime or nighttime feeding cycles generate more displacement and to what degree resting sites or resources, such as waterholes, create centrality in meso-movement patterns, we began by standardizing individuals’ daily fixes to roughly 6 h intervals represented by a fix at “dawn” (07:00 ± 2:30), “midday” (13:00 ± 2:30), “dusk” (23:00 ± 2:30), and “midnight” (23:00 ± 2:30). Because of the considerable variation among collar fix rates, we liberally interpreted a fix as within 2.5 h of each nominal time of interest. Additionally, natural variation in sunrise and sunset times throughout the year (roughly from 6:20–7:30 for sunrise and 18:25–19:35 for dusk) was easily captured within the ± 2.5 h buffer to our designated dawn and dusk fix times. If there were multiple fixes within the interval, we selected the one closest to the hour of interest. For example, a rhino with a fix at 5:45 am and no other before 9:30 am would have this fix recorded as a “dawn” fix for that day. Using this standardized dataset, we then calculated the displacement between each ≈12 h interval (e.g., dawn to dusk), and ≈24 h interval (e.g., dawn to dawn). Since actual fix intervals were allowed to vary around the intervals of interest (i.e. 12 h and 24 h), we standardized each displacement measure by the exact time between consecutive fixes, using a measure of displacement per hour.

For initial analyses, we selected 9 individuals with at least 30 days consistent fixes at likely foraging hours for dawn and dusk. We then plotted the 24-h displacement time series (calculated from dawn to dawn) for each individual and tested for periodicity using a Fisher’s test for periodicity in short time series [35]. Additionally, we built and visually inspected density plots for 24-h displacement measurements as measured from dawn to dawn and dusk to dusk. We hypothesized that daily displacement by rhino individuals would demonstrate a bimodal frequency distribution indicating support for day-to-day movement patterns that include high rates of short-term residency interspersed with occasional long distance movements to new parts of the greater home range. To further investigate our hypothesis, we repeated our analyses using the complete data set: i.e., requiring no daily consistency and calculating distances for any complete pairs of points. With this data we additionally calculated displacement at an ≈6 h interval (e.g, dawn to midday) to allow us to investigate resting cycle displacement for comparison. Since rhinos are known to visit waterholes daily to drink, mostly soon after dusk or before dawn [14], we hypothesized that dusk and dawn displacement would be more conservative than resting period displacement. In other words, we expected that daily recursion to waterholes, tied to hydration demands, would result in a high frequency of smaller displacement measures. To further evaluate our hypotheses regarding waterhole use, we calculated the frequency of points from each time of day within a 250m radius of known waterholes in Etosha National Park. Pairwise Kolmogorov–Smirnov tests with Bonferroni correction were used to test for differences across empirical 24-h displacement distributions. Daily displacement patterns were additionally investigated across wet (November - March) and dry (April - October) seasons and between the sexes; Seasons were determined using rainfall data sampled from 7 sites within Etosha National Park in 1981–2013 (see Additional file 1: Appendix B).

### Biweekly recursion analysis

For optimal foragers, patch visitation and revisitation on spatially heterogeneous landscapes is thought to be driven, in part, by the productivity cycle. Feeding in a patch leads to local resource depletion, potential individual satiation, and optimal patch-leaving decisions [36, 37]; after the animal leaves a patch, environmental resources replenish incentivizing revisitation [38]. It’s this visitation cycle, when done by rhinos or other megaherbivores, that is thought to be a mechanism for browsing lawn maintenance and prevention of canopy growth. To estimate how landscape structure and productivity may influence rhino movement and short-term patch recursion, we downloaded 16-day, 250 m^2^ resolution, composite images from MODIS satellites using NASA’s earthdata search for all 16-day intervals in 2011–2018 and extracted the NDVI product layer. The Normalized Difference Vegetation Index (NDVI), is a commonly-used, remotely-sensed measurement of productivity and an index of canopy cover. Previous studies have shown a linear relationship between NDVI and percentage vegetation cover, with increased correlation to canopy structure (i.e green biomass, green leaf area index) in areas of sparse canopy [39, 40]. Furthermore, previous work investigating NDVI in Namibian savannas suggests minimum NDVI may be a suitable metric for woody cover across our study area [41].

To estimate patch recursion, we divided individuals’ trajectories into 16 day intervals aligned with MODIS satellite collection periods in each year of sampling. Using the tlocoh and tlocoh.dev packages [42, 43], we built time use grids for each 16 day trajectory where at least 1 fix was recorded per day for at least 15 days of the interval (as a means of removing any trajectories with large gaps that could bias our results). These time-use grids were built across the complete extent of each individuals’ relocations during each interval and calculated two statistics: the number of separate visits (nsv) to each cell and the mean locations per separate visit (mlsv) in each cell, which estimate recursion and duration of visit respectively [44]. mlsv can be considered a duration metric: mean locations per separate visit is calculated as the average number of individual time points for which a rhino was in a given cell during each visit (as defined by the inter-visit gap). For this analysis, locations were considered separate visits if more than 12 h passed between locations within the same grid cell and each grid cell has an area of 1 km^2^. This spatial resolution was chosen given the relative temporal coarseness of our relocations and the fact that foraging groups for large herbivores can span large areas.

Overlaying the constructed time-use grids on to the contemporaneous MODIS imagery for each available trajectory, we used R packages sf and velox [45, 46] to extract the average, median, minimum, maximum, and standard deviation of NDVI values for each 1 km^2^ grid cell. We visualized the relationship between nsv, mnlv, and mean NDVI using the ggplot2 package in R [47].

### Annual recursion and home range analysis

To understand how meso-scale rhino movements may translate into home ranges and landscape level spatial-use patterns, we need to examine recursion over a much larger temporal and spatial scale. To begin, we identified 6 individuals within Etosha National Park with consistent fixes for a complete year, from April 2012 to April 2013. Using the same T-LoCoH method as above, we built time use grids for all 6 individuals over the course of their entire trajectories (including fixes beyond the April 2012–2013 interval) identifying separate visits using an inter-visit gap (ivg) of 7 days (as compared to ivg = 12-h used above). Using a spatial join, we then identified each separate visit within each individuals’ time use grids and measured the time to return in days between all visits for all grid cells receiving at least 3 visits within the year. Grid cells including a known watering hole were removed from this analysis in order to investigate patterns of recursion to presumed foraging sites independent from watering hole use.

As the annual range sizes of our 6 individuals varied widely, we sought to explore how size of range influenced recursion patterns. Are individuals in a constricted range using all parts of their range more frequently than individuals in larger ranges? Are they returning to select areas more or less intensively than their large-range conspecifics? Does range size affect the number of patches, or proportion of an individual’s range, an individual uses most intensively? To evaluate these questions, we calculated the mean, median, and standard deviation of the number of separate visits across cells that received at least two visits within the year for each individual. Additionally we calculated the proportion of sites revisited by dividing the number of cells with at least 2 visits by the number of cells with at least 1 visit by the same individual. Finally, we tallied the number of cells for each individual whose number of separate returns was within the top quartile of nsv values observed for that individual. Using Pearson’s correlation coefficient, we evaluated the linear relationship between range size (measured as the number of grid cells visited at least once within a year’s trajectory) and the mean number of returns to cells, and the standard deviation in number of returns to cells. Additionally we evaluated the relationship between range size and the proportion of cells revisited.

Finally, once again considering all 59 individuals available, to understand how productivity of resources within an individuals’ range may inform the size of a range used or needed, we built polygons representing the 90% isopleths of utilization distributions estimated using *k*-type local convex hull estimation (*k*-LoCoH), a conservative non-parametric estimator of home range especially good at identifying ranges including hard boundaries or unused areas (e.g., the Etosha Pan) [48, 49] (but see Additional file 1: Appendix D for comparison to an alternative estimator). Isopleths were built on the 16 day intervals identified to align with MODIS imagery for all trajectories including at least one fix per day for 90% of the interval. We then extracted the mean and variance of greenness (viz., greenness=NDVI from MODIS imagery) within each intervals home range using the velox and sf packages in R [45, 46]. To evaluate the relationship between area and greenness, we fit a generalized linear mixed model using packages lme4 [50] to handle our unbalanced, longitudinal data, including repeated measures across individuals. Area measurements were log-transformed before analysis in order to better meet the assumptions of linear regression.

## Results

### Twenty four hour displacement cycles

**Fig. 3:**
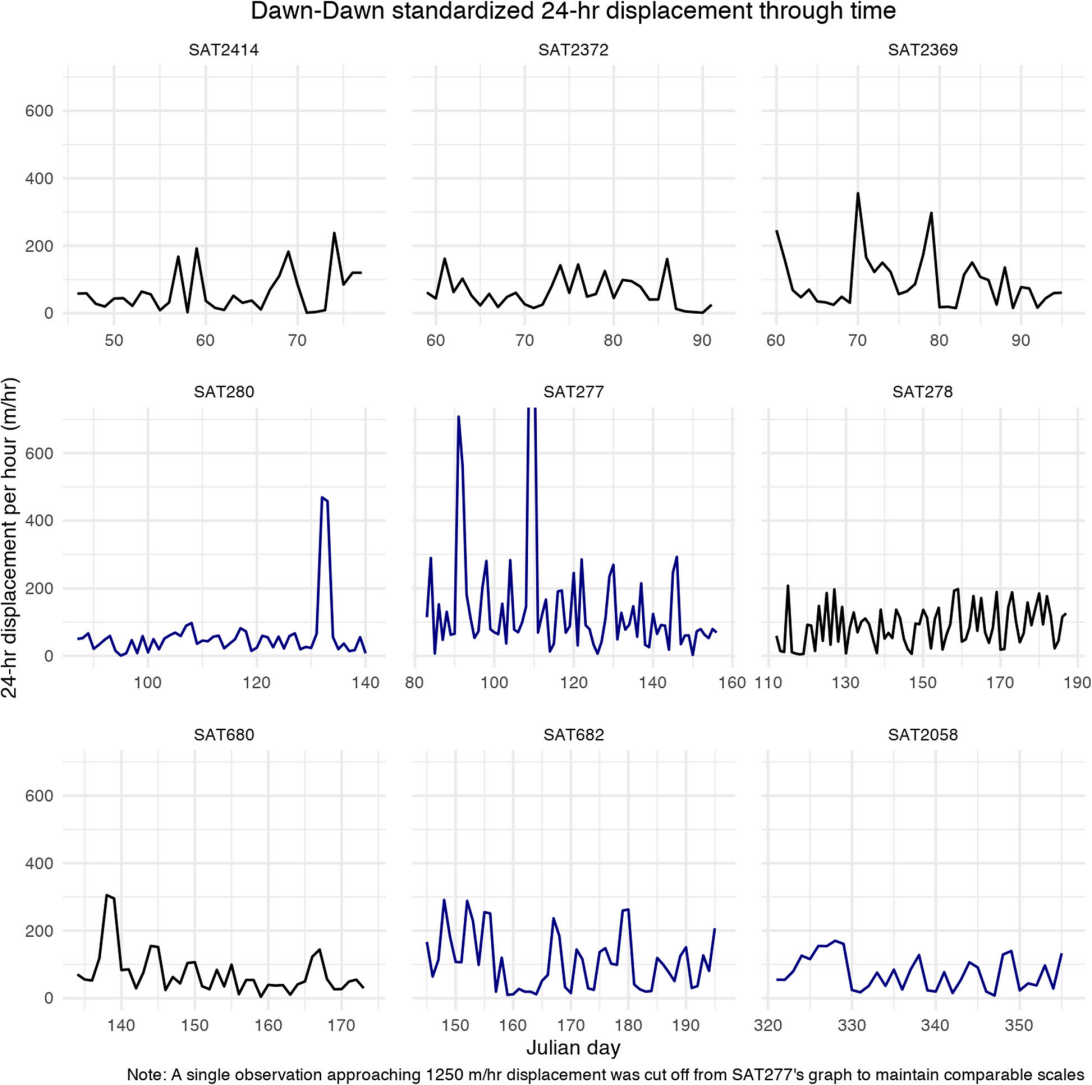
Standardized 24-h displacement time series plot for individuals with consecutive fixes. Dark blue is used to differentiate the displacement patterns of male rhinos from that of females (plotted in black). Note that the individual plots are arranged so that Julian dates get larger right to left and down the columns to aid the reader in evaluating seasonal trends

**Fig. 4:**
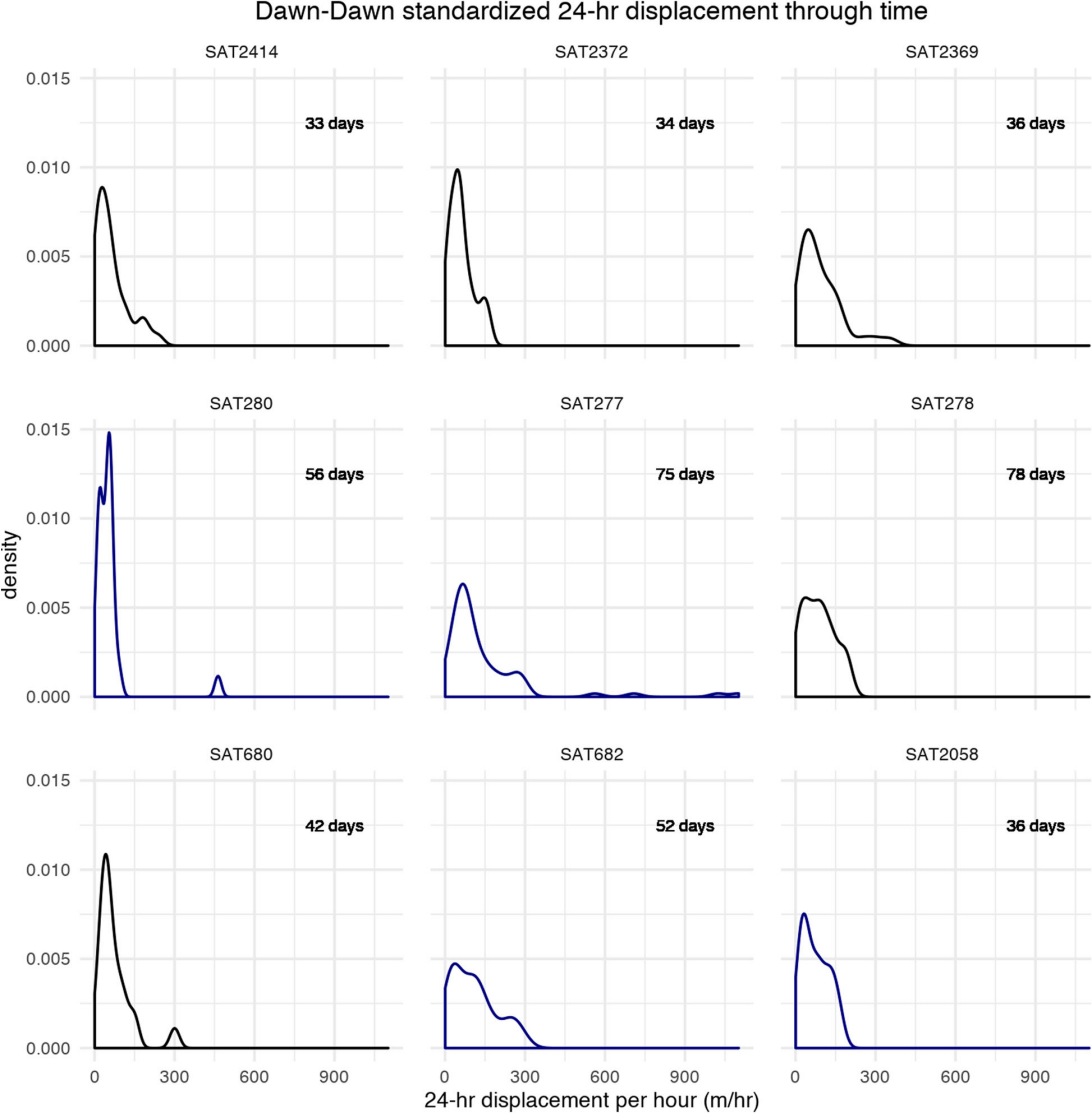
Density plots of standardized 24-h displacement across individuals with consistent fixes. The order of plots reflects that in Fig. 3 and the dark blue lines again are used to distinguish male rhinos. The bimodality in some plots offers weak support for the notion of short movements interspersed with occasional long movements within and between areas of the home range

Displacement time-series plots (*x*-axis, Julian day), for 9 individuals with consistent 12-h fixes for at least 30 days, provided some visual evidence for our hypothesized pattern of a series of short displacements followed by occasional large displacements; however, there was little consistency across individuals and no visible pattern across time of year or sex (Fig. 3). Fisher’s g test for periodicity failed to reject the null hypothesis of no periodicity (at alpha =.05) in all but one individual, SAT2372. Across the individual density plots for these individuals, only minor bimodality is seen in some individuals standardized 24-h displacement (Fig. 4).

**Fig. 5:**
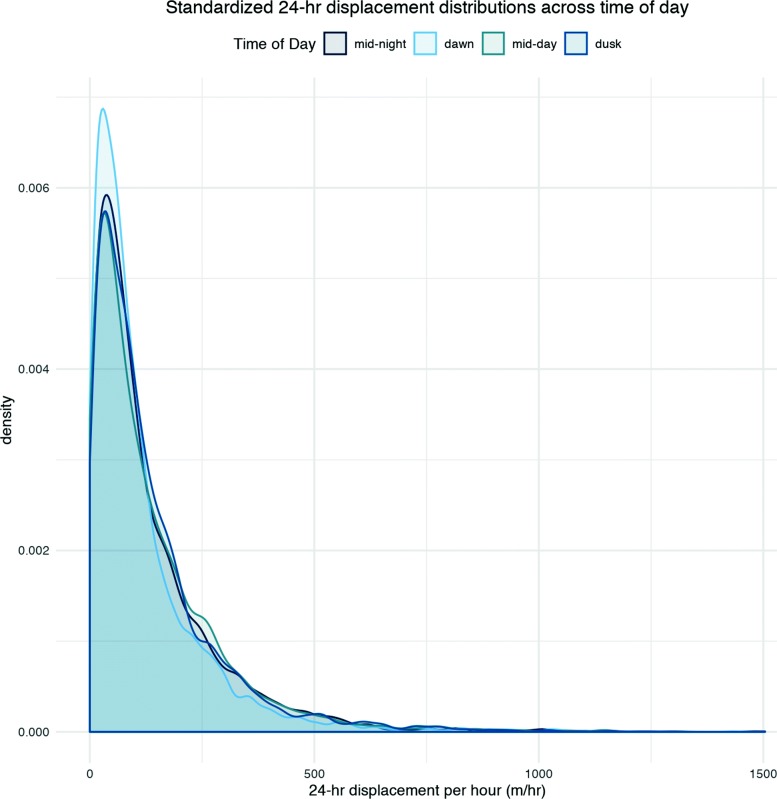
Density plots of standardized 24-h displacement across 4 regularly spaced diurnal starting times. Dawn-to-dawn times are significantly different from the other three (*p*<0.05 see text for details), driven by a higher frequency of shorter displacements

Investigating 24-h displacement measures, across 4 different starting times (dawn, midday rest period, dusk, midnight rest period), revealed that the dawn-to-dawn displacements where on average smaller than displacement measures starting at the other three times of day (midnight, midday, and dusk), which where similar among the themselves (Fig. 5). Pairwise KS-tests between each time of day displacement distribution were run and *p*-values corrected for multiple testing using the Bonferroni correction. In all tests, we failed to reject the null hypotheses that mid-day, mid-night, and dusk samples all came from the same underlying distribution; however, we rejected the null hypothesis that dawn samples came from the same underlying distributions as other times of day (*p*<.05; see Additional file 1: Appendix A for detailed results). This pattern may indicate that, in general, rhinos are staying within the same area of their home ranges for days at a time, and possibly returning to the same general area days in a row, especially during morning foraging hours.

Investigating the pattern of 24-h displacement across sexes revealed that, in general, males moved larger distances than did single females or females with calves, but the dusk/dawn pattern held. Investigated across seasons, we would expect that the increased availability of resources in the wet season would erase this effect by reducing the need for long distance movement to find productive resources. As expected, across midnight, midday, and dusk points, more shorter 24 h movements occurred in the wet than dry season. Twenty-four-hour movement patterns at dawn remained consistent across seasons indicating that dawn-dawn displacement is less water-dependent. For more information, see Additional file 1: Appendix B. When investigating the time of day of relocation points nearest to watering holes, dusk had the highest number of relocations found within 0.25 km of a watering hole (*n*=179; see Additional file 1: Appendix E for results under alternative buffer distances). Midday and mid-night followed (*n*=116 and *n*=123 respectively). Dawn fixes within range of a watering hole were least frequent (*n*=32) supporting previous research that rhinos predominately drink after dusk but also indicating that drinking before dawn is less common. The relationships confirm the importance of the hydration cycle to daily movement with dawn foraging away from water in favoured feeding areas, dusk foraging nearer water and resting site in between.

### Biweekly recursion analysis

**Fig. 6:**
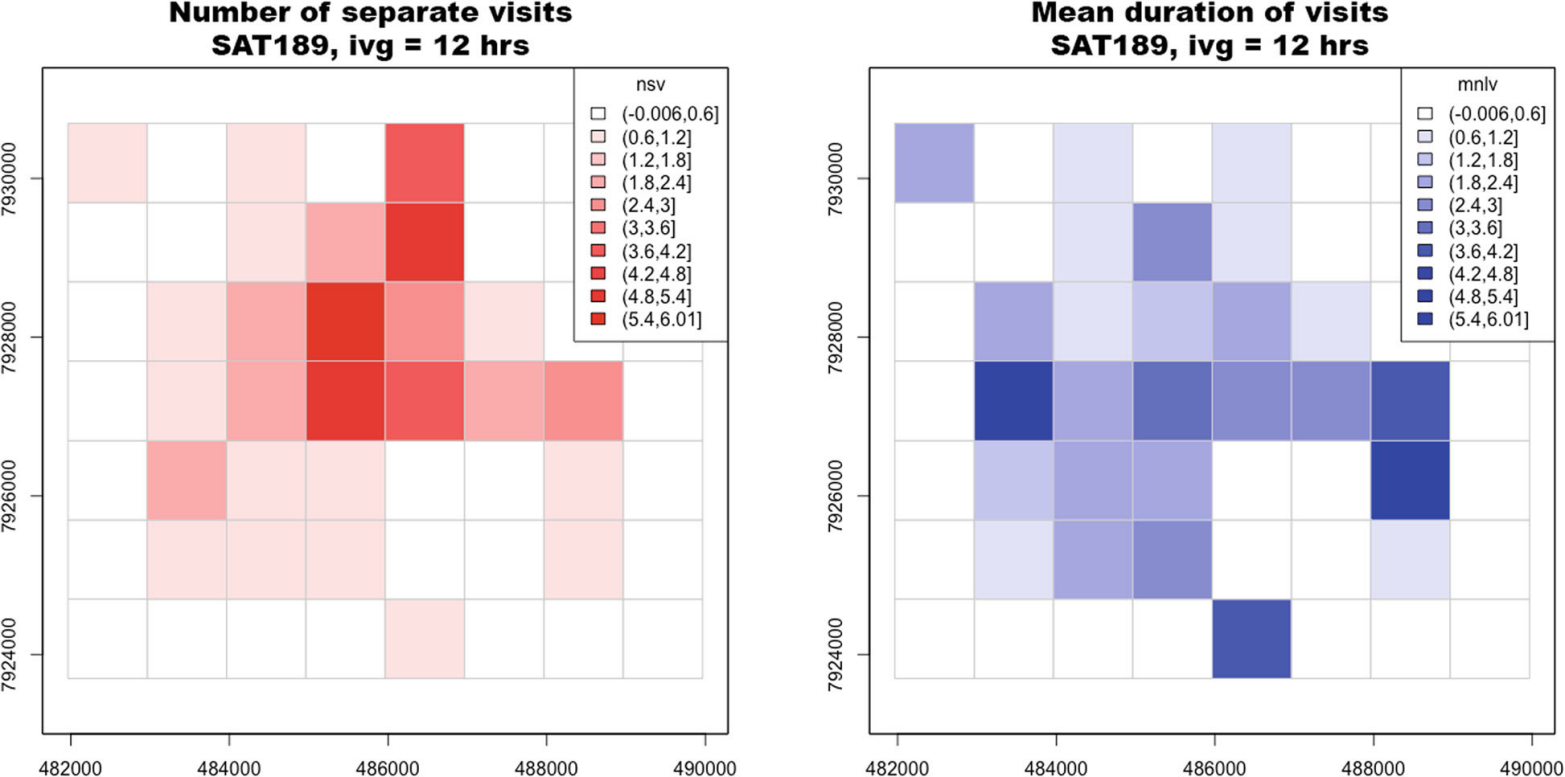
Example time-use grid plots for rhino SAT189. Side-by-side plots illustrate two time-use metrics for a single 16-day interval for individual SAT189. Left panel: the number of separate visits (nsv) to each grid cell (1 km^2^ areas with an intervisit gap of 12 h apart). Right panel: the mean number of locations per visit (mnlv) to each grid cell

By splitting trajectories into 16-day intervals and ensuring at least 1 fix per day on 90% of the days, we obtained 480 unique coarse-grained 16-day trajectories, across 48 unique individuals. Time-use grids for each unique 16-day trajectory showed different patterns for those cells that had high recursion rate (high nsv) and those that had long visits (high mlsv) (Fig. 6). This likely indicates that features or regions exist that rhinos regularly return to but do not stay long and conversely places where they may not visit frequently but upon arrival stay for extended periods. It’s worth noting that while our methods removed intervals with large gaps (>24 h), to maximize the number of individuals included, fixes were not regularized or interpolated before building time-use grids. Therefore, estimates of nsv and mlsv may be underestimated for some intervals and individuals and should be interpreted as estimates of the lower bounds. Across all grid cells used by the 48 individuals and across all intervals (*n*=10733), the average number of separate visits (nsv) to each grid cell was 1.86 (*σ*=1.50) and the average visit duration (mnlv) was 1.52 (*σ*=1.20 fixes).

**Fig. 7:**
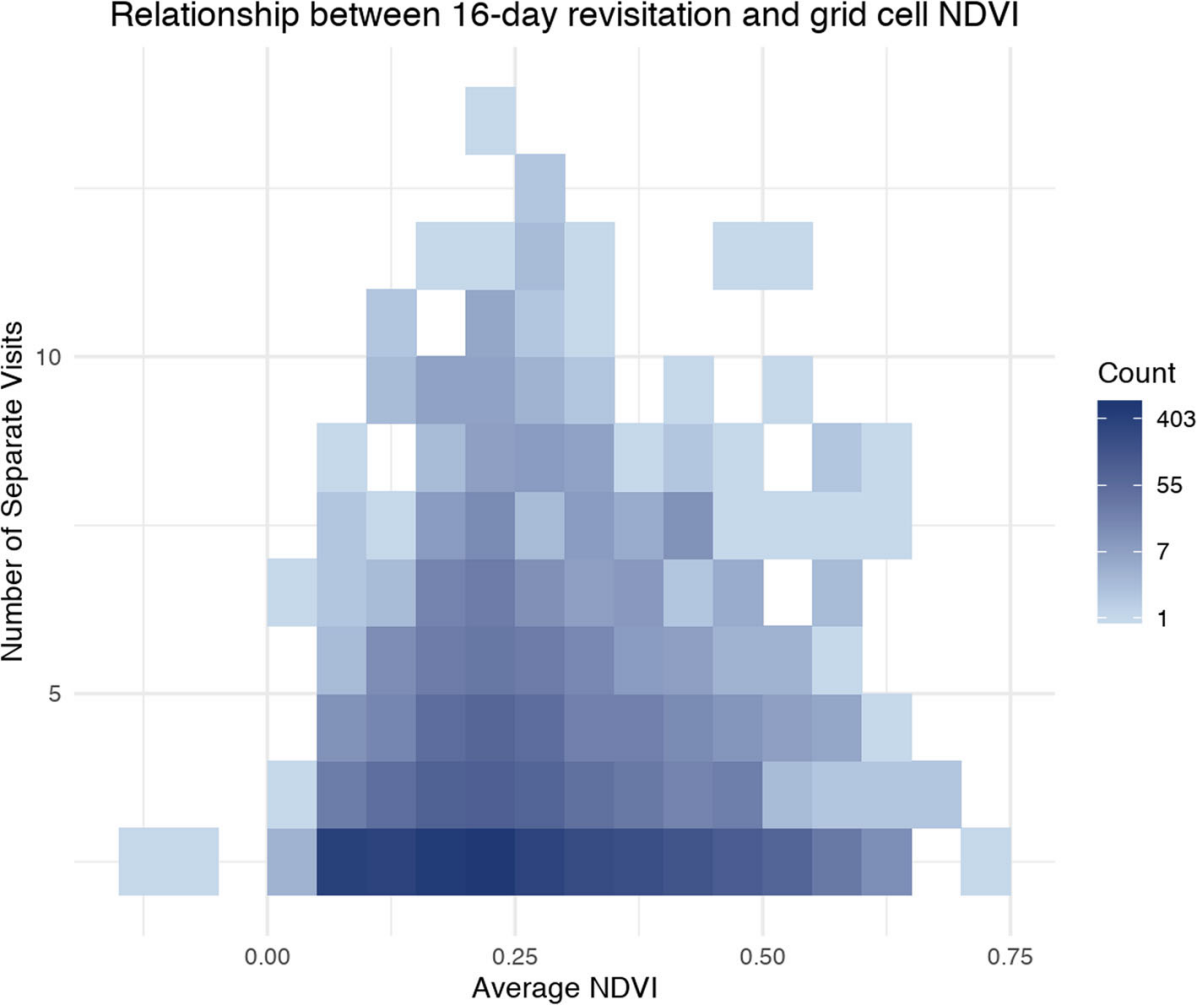
Relationship between 16-day revisitation and grid cell NDVI. A heat map showing the density of points along axes of number of separate points and mean NDVI values. The color scale is log transformed to better visualize the variability at the lower end of the nsv measure. Note that the highest density of grid cells have 2 visits and a mean NDVI of ≈0.25. This plot does not include grid cells not visited or returned to, i.e. nsv <2

Our plot of number of separate visits against mean NDVI extracted for each grid cell visually shows a hill-like relationship with NDVI whereby areas with mid-range NDVI values are most revisited (Fig. 7). The duration statistic, mnlv, shows a similar pattern. The mean NDVI of all cells visited at least once equaled 0.23 ($\bar {x} = 0.23$, *σ*=0.02); Globally, NDVI values of 0.2 to 0.3 generally reflect shrub or grassland ecosystems which is consistent with the study site [51]. See Additional file 1: Appendix C for further details on the background distribution of NDVI in the area and a comparison of linear and quadratic model fits.

### Annual recursion and home range analysis

**Fig. 8:**
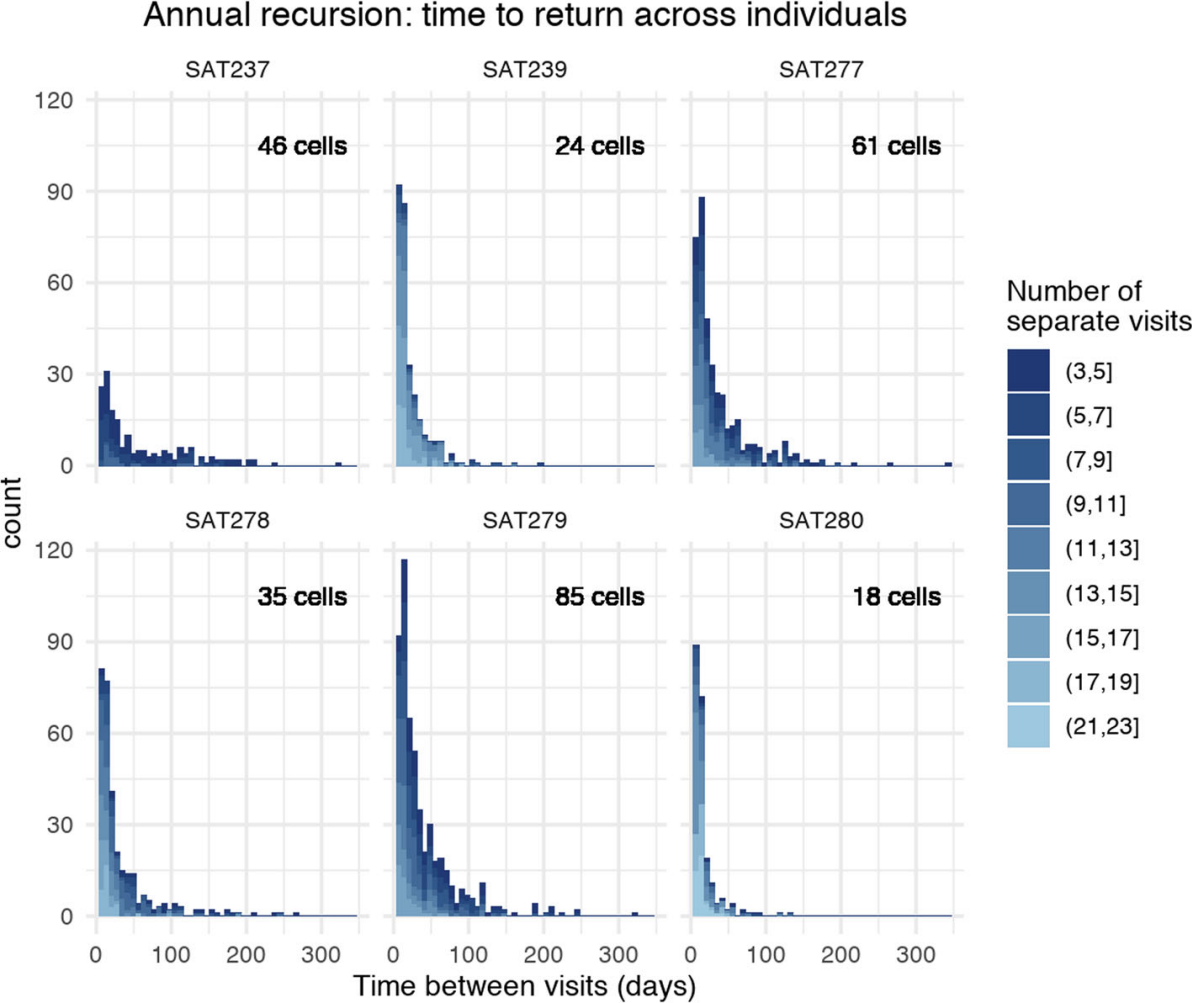
Side-by-side histograms display the distribution of time between visits to grid cells for each individual over the course of a year. The bin width of each bar is 7 days. Lighter colors indicate returns to grid cells with higher numbers of returns. The number of visited grid cells in each individuals range is included in the top-right corner of each plot to highlight the variability in recursion patterns correlated with range size. Across all individuals the most common time to return is within 8–21 days (the first 2 weeks), but all individuals see at least some returns months apart. Individuals with smaller ranges tend to have higher rates of return and shorter times between returns. Individuals with larger ranges have much longer tails to their distributions, potentially showing support for seasonal returns to different areas of their home ranges

Our investigation of time-to-return between visits on an annual scale showed high variation. Rhinos most commonly returned to sites with high recursion rates (nsv >=3) within 8–21 days of the last visit; however, across all individuals, some returns occurred months apart (Fig. 8), particularly in individuals with larger ranges. When examining recursion across the year, we found that high levels of recursion were influenced by the size of the overall range of the individual, with longer times between returns being more common among individuals with larger ranges. Two out of six of our investigated individuals had notably constricted ranges; in, at least, one case due to obvious environmental barriers within the range. Range size was also strongly negatively correlated with median number of separate visits (*r*=−0.97, *p*-value = 0.00, 95% CI: -0.10 - -0.76) and standard deviation of number of separate visits (*r*=−0.85, *p*-value = 0.03, 95% CI: -0.98 - -0.14), indicating a relationship between smaller ranges and higher revisitation to grid cells overall but also support for higher variation among grids cells of smaller ranges.

The proportion of revisited cells was also negatively correlated with range size (*r*=−0.649, *p*-value = 0.16, 95% CI: -0.96 - -0.34) but appeared skewed by a single point (without SAT280: *n*=5, *r*=−0.96, *p*-value = 0.01, 95% CI: -0.98 - -0.50). Interestingly, the proportion of visited grid cells that had the 25% highest number of separate returns for each individual was fairly stable across all individuals and range sizes ($\bar {x} = 0.22$, *σ*=.03). Upon further investigation, our outlier SAT280 was found to have a particularly concentrated range around a single watering hole with few, irregular, excursions and exploratory movements outside of the immediate surrounding area; this individual recorded the highest revisitation values among all 6 rhinos.

Finally, investigating the relationship between range size and productivity, we found that (log-transformed) area of home range was inversely correlated with mean NDVI values within the short-term ranges (intercept = 16.25±0.19, *β*=−1.67±0.54).

## Discussion

Our results confirm that black rhinos make recursive movements at daily, biweekly, and annual scales; but, for the first time, we have measures for the intensity of these activities among black rhino in northern Namibia and the relationship between recursive movements and spatial resource heterogeneity within rhinos’ home ranges. Daily displacement measurements did not strongly support long held assumptions about the potential mesoscale movement of rhinos. Instead they raise new ideas and questions about the daily movement cycle, especially with regards to differences between the dusk-to-dusk and dawn-to-dawn displacement patterns. Our investigation of recursion at the biweekly scale suggests that individuals are returning most frequently to patches with mid-range NDVI values; which, perhaps, is evidence of preference for intermediate shrub environments. We found a strong negative relationship between short-term range size and NDVI indicating that individuals in smaller ranges incorporate higher NDVI on average than individuals with larger range estimates. Recursion to patches across rhino annual home ranges, most often occurred within 2–3 weeks of the last visit, although we also found evidence of seasonal recursions (months apart), particularly in individuals with larger ranges.

Before investigating daily displacement of rhino movement, we hypothesized that daily displacement by individual rhino would produce a bimodal frequency distribution, thereby indicating support for day-to-day movement patterns that include regular short intra-patch movements interspersed with occasional (e.g., weekly or bimonthly) long distance inter-patch movements to new parts of their larger home ranges. Our results, however, showed only weak support for this hypothesis in some individuals, but no bimodal pattern at a monthly or shorter level. Instead, we found that 24-h displacement measures have a distinct daily cycle to them, with daily recursion more likely at dawn, whereas dusk-to-dusk and midday and midnight resting cycles produced greater displacement. In contrast to our hypothesis that movements should be more conservative at both dusk and dawn as they are both feeding periods, we found only dawn-to-dawn displacements were conservative.

Rhinos are known to visit waterholes daily to drink, mostly soon after dusk or before dawn. Thus, we also hypothesized that dusk and dawn displacement would be more conservative than resting period displacement. However, in Etosha National Park, where we had coordinates of known watering holes, dawn fixes were among the least likely to be found near watering holes when compared to the other 3 times of day. Also, daily dawn displacement was significantly smaller than at other times of the day. Lastly, comparisons in 24-h displacement between wet and dry seasons revealed a reduced dusk-to-dusk and midday and midnight displacement suggesting, as expected, that when resources are more plentiful, shorter movements are more viable or attractive. However, dawn-to-dawn displacement was similar among the seasons.

These observations of 24-h dawn displacement and other times of day in the different seasons inspires new questions and hypotheses about rhino movement. Rhino appear more likely to adopt a recursive strategy to favoured foraging patches in the morning than night-time foraging periods which are more influenced by the need to rehydrate after dusk. The 24-h displacements we observed may illustrate a trade-off between optimal patch foraging and rehydration where waterholes and favoured forage are distant from each other. Further investigations of the role watering holes and a daily hydration cycle play in daily rhino movements are necessary to obtain a better understanding of this dawn-to-dawn conservative movement phenomenon, especially in cases where complete knowledge of water source locations are available. Furthermore, we suggest that investigating distributions of 24-h displacement is a useful way of analyzing intermediate-scale movement. In addition, with appropriate interpretation, it is a way to usefully analyse low resolution, gappy animal movement data. Given that much of rhino relocation is commonly gappy and coarse, our approach could help researchers further probe long held intuitions about the way rhino—as well as species such as hippo, with similarly challenging or underutilized data sets—move between and use different areas of their home range.

By investigating biweekly recursions, we were able to link intermediate movement patterns with the finest available temporal resolution for an index of dynamic spatial heterogeneity, NDVI. Our results demonstrate that patch recursion occurs even within as short a time scale as 16 days. Further, the most frequently returned to patches reflect a preference for mid-range NDVI of around 0.25. This range is consistent with global expectations of grassland and shrub ecosystems (higher values would generally correlate with more canopy cover and forest greenness); but, it is interesting to hypothesize why rhinos may select for median rather than maximum available productivity within this system. The intermediate disturbance hypothesis [52] predicts that some moderate level of herbivore feeding provides the spatially and temporally heterogeneous conditions for greater biodiversity and increased productivity of favoured forage. Given this hypothesis, we should expect that rhinos’ foraging would spur productivity, which in turn may attract recursion. If such productivity dynamics are accurately reflected in NDVI, mid-range NDVI patch selection may reflect an intermediate greenness value maintained in the most preferred and hedged browse (like grazing lawns) with the highest rates of recursion. Of course NDVI is only one measure; and, in this case, perhaps an imperfect one. Further study with the aid of LIDAR or other imagery that provides vegetation height and structure data would help to better understand the foraging environments rhinos use intensively.

Interestingly, although home-range size varied among individual rhino, we found that the proportion of grid cells in their range that each frequently revisited did not vary. This would be true if rhino adjusted their range size to include some satisfactory minimum number of feeding patches. In lower quality ranges, patches are sparse and so the range must be larger. In addition, rhino in larger ranges had the longer recursion intervals and lowest recursion frequencies. These patterns are consistent with the idea that feeding patches support more frequent recursion over shorter time frames because they are in more productive habitat. It also supports the idea that rhinos use recursion to engineer productivity in their ranges. Both ideas can be true in a positive feedback between habitat quality and recursive feeding. Further meso-scale rhino movement studies are needed to fully explore these ideas and test whether rhinos are in fact engineering their habitat or just responding to its resources.

An understanding of intra-home-range movement is crucial to bridge existing research at the fine (step-by-step) and macro (home-range) movement scales. By analyzing recursion requiring 7 days between unique visits over an annual cycle, we were able to identify patches within the home range of prolonged, and possibly sustained, value to rhinos over the course of the year. Our results offered new insight into how range size may affect resource use within an animal’s range long term, an especially relevant topic given that the surviving populations of rhinos are often in small, fenced, and sometimes isolated reserves or ranges. Our analysis of grid cells with known watering holes provided evidence that these cells often received a very high frequency of separate visits with low average duration at a 7-day inter-visit-gap resolution, although our sample size was too small for this evidence to be definitive. Additionally, though our analysis included all known watering holes within the park, it is likely that some seasonally available, or small, unmarked waterholes went unidentified. Our results suggest, however, that our scale of recursion analysis can become an effective tool for identifying locations of previously unknown watering holes. Future analysis is needed to investigate how long-term recursion patterns and time to return track directly with productivity and may change in the wet versus dry season. If recursion is driven by resources and productivity, one might hypothesize given the increased resource availability and productivity during wet seasons that time to return would be significantly shorter than during the dry season where biomass regeneration is slowed.

## Conclusions

These results are a rare glimpse into meso-scale movement patterns of the black rhinoceros across a majority of its remaining range in Namibia. The black rhino population sampled here is the third largest in Africa and the only viable population of *Diceros bicornis bicornis*. The endemism of this unique sub-species and the rhinos’ unique adaptation to the arid habitat in the west makes it all the more crucial to conserve. However, the impact on global biodiversity of conserving the black rhino goes well beyond the conservation value of a single species. Globally, megaherbivores (>1000 kg [24]) support an extraordinary amount of biodiversity as ecosystem engineers. Through their feeding behavior and long distance migration and dispersal, megaherbivores maintain open landscapes by reducing tree cover, transport seeds and nutrients, and significantly influence the species composition and carbon storage in the ecosystems they inhabit [28, 53–56]. A better understanding of their recursive movement patterns, particularly at the meso-scale, is crucial for understanding and conserving this species and the unique ecosystems they help fashion.

## Supplementary information

**Additional file 1** Appendices A - E including supplemental analyses and results.

## Data Availability

The datasets generated or analyzed during the current study are not publicly available in order to protect the locations and resources of a critically endangered species but may be made available from the corresponding author upon reasonable request. Cleaned analysis scripts are available on GitHub and archived with Zenodo (10.5281/zenodo.2783972). The MODIS imagery analyzed is freely available from NASA.gov. The global ecoregions shapefile used in Fig. 1 is freely available from http://ecoregions2017.appspot.com.

## Abbreviations

GPS: Global positioning system
LIDAR: Light detection and ranging
MODIS: Moderate resolution imaging spectroradiometer
NASA: National aeronautics and space administration
NDVI: Normalized difference vegetation index
T-LoCoH: Time-method local convex hull
UHF: Ultra high frequency
