# Supplementary material for: Mesoscale movement and recursion behaviors of Namibian black rhinos
Source: Mov Ecol. 2019 Nov 9;7:34. doi: 10.1186/s40462-019-0176-2 (PMC6842456; doi:10.1186/s40462-019-0176-2)
Supplement: Supplementary file 1 — Additional file 1 Appendices A - E including supplemental analyses and results. [file 40462_2019_176_MOESM1_ESM.pdf]

Appendix A: Pairwise Two-sample KS Test p-values with Bonferroni Correction

To test whether the distribution of dawn-dawn displacement differed significantly from distributions of displacement measured at other times of day, we implemented a pairwise KS-test with Bonferri correction for multiple testing. In all tests, we failed to reject the null hypotheses that mid-day, mid-night, and dusk samples all came from the same underlying distribution; however, we rejected the null hypothesis that dawn samples came from the same underlying distributions as other times of day ($p < .05$; Table 1)

Table 1 Results from Pairwise KS test with Bonferroni correction

Sample Pair	Bonferroni corrected p-value
dawn:mid-day	0.000
dawn:dusk	0.000
mid-night:dawn	0.000
mid-night:dusk	0.057
mid-night:mid-day	0.726
mid-day:dusk	0.074

Appendix B: 24-hr displacement: Seasonal and group effects

B.1 Seasonal behavior

Wet and dry seasons were determined using monthly rainfall data for the region (Fig. 1).

Comparisons in 24-hour displacement between wet and dry seasons revealed a reduced dusk-to-dusk and midday and midnight displacement (Fig. 2). This suggests that when resources are more plentiful, shorter day-to-day movements may be more viable or attractive, i.e. individuals may persist in the same relative area of their home ranges for longer times.

This pattern held using the larger dataset, and was seen across all times of day, suggesting that availability of resources may support more conservative daily movements (Fig. 3).

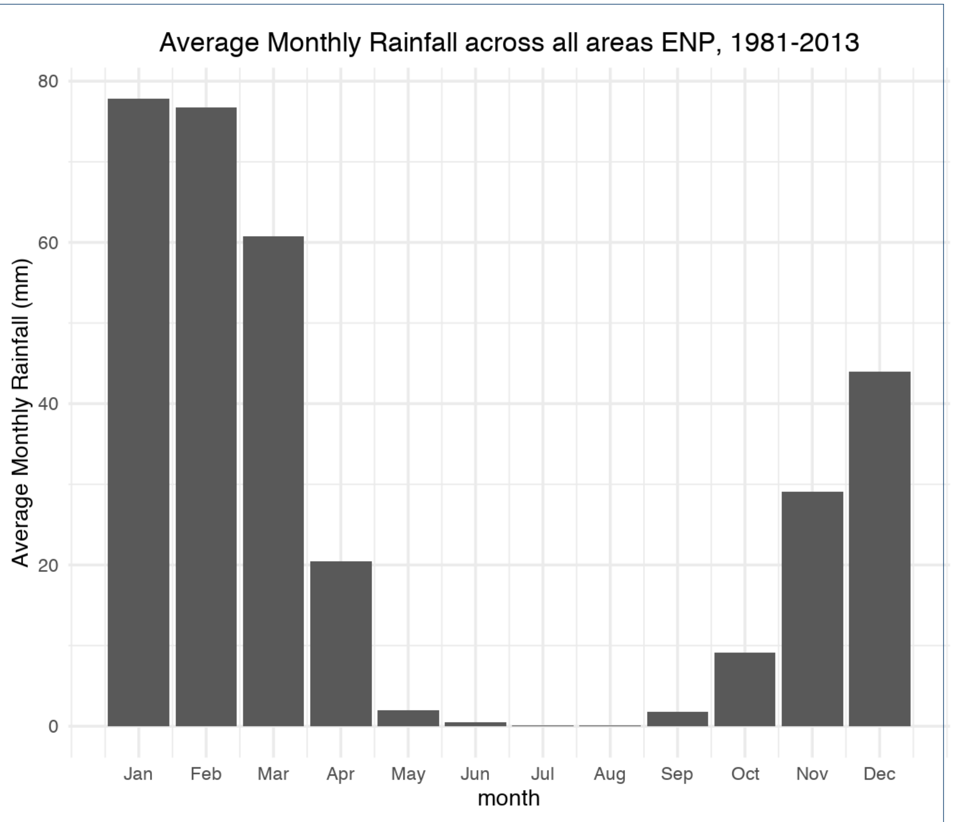

Figure 1 Average monthly rainfall in Etosha National Park (1983-2012. Using daily rainfall data collected from gauges across Etosha National Park from 1983 - 2012, monthly averages are calculated to support delineation of two seasons: the wet season (November through April) and the dry season (May through October).

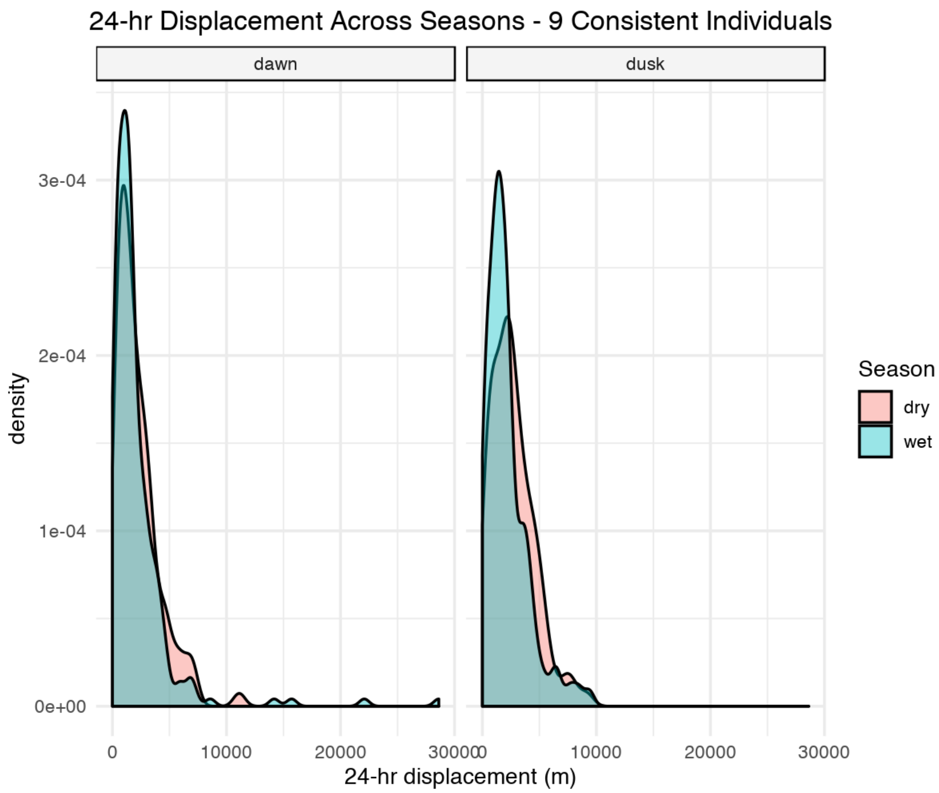

Figure 2 Seasonal dawn and dusk 24-hour displacement. Calculated from 9 individuals with consistent fixes for at least 30 days, shorter, more conservative 24-hr movements are more common at both dusk and dawn during the wet season. Displacement at dusk during the wet season is reduced dramatically to reflect a similar pattern as seen in dawn-dawn displacement during the dry season.

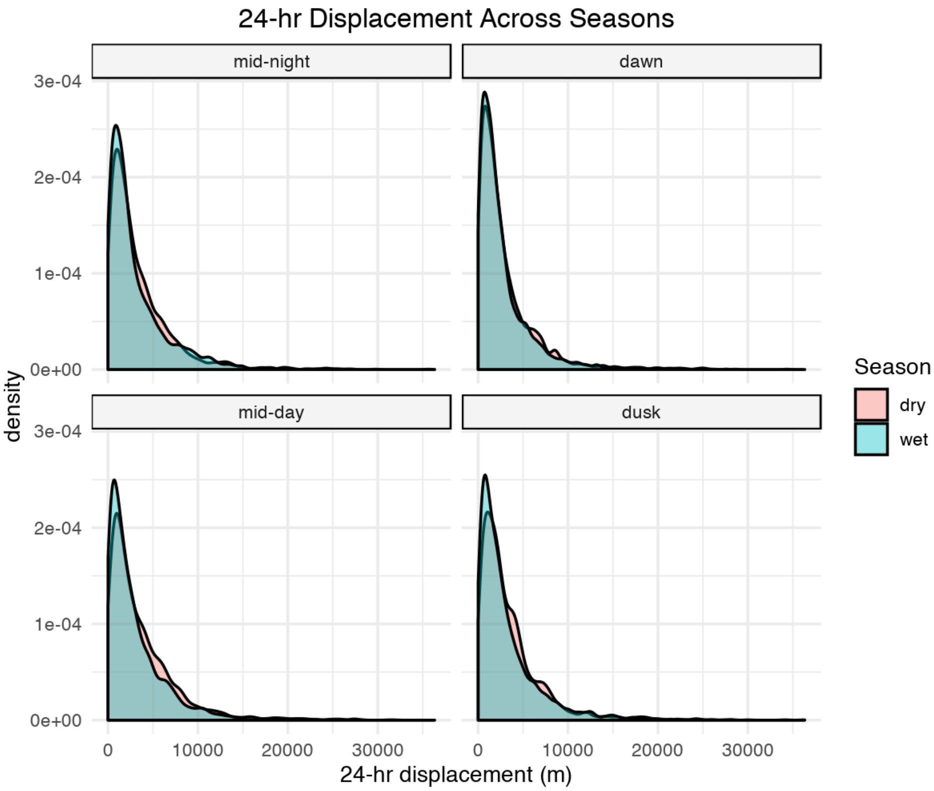

Figure 3 24-hour displacement across time of day and season. The frequency of short distance displacement is increased across all times of day in the wet season, suggesting that the availability of resources may support or allow for more conservative daily movements similar to those seen dawn-dawn year round.

B.2 Life History

Solo females and females with calf had very similar 24-hour displacement distributions, with females with calves exhibiting the highest frequency of conservative dawn-dawn movements. Males demonstrated the same relative pattern of dawn-dawn displacement being most conservative when compared to displacements measured at different times of day but had a higher frequency of long distance displacements overall than did females (Fig. 4). Greater male, than female, displacement is expected given the territorial and breeding activities of bulls. Male ranges are ordinarily regarded as territories and a successful bulls territory typically overlaps more than one breeding females territory (Owen-Smith 1988).

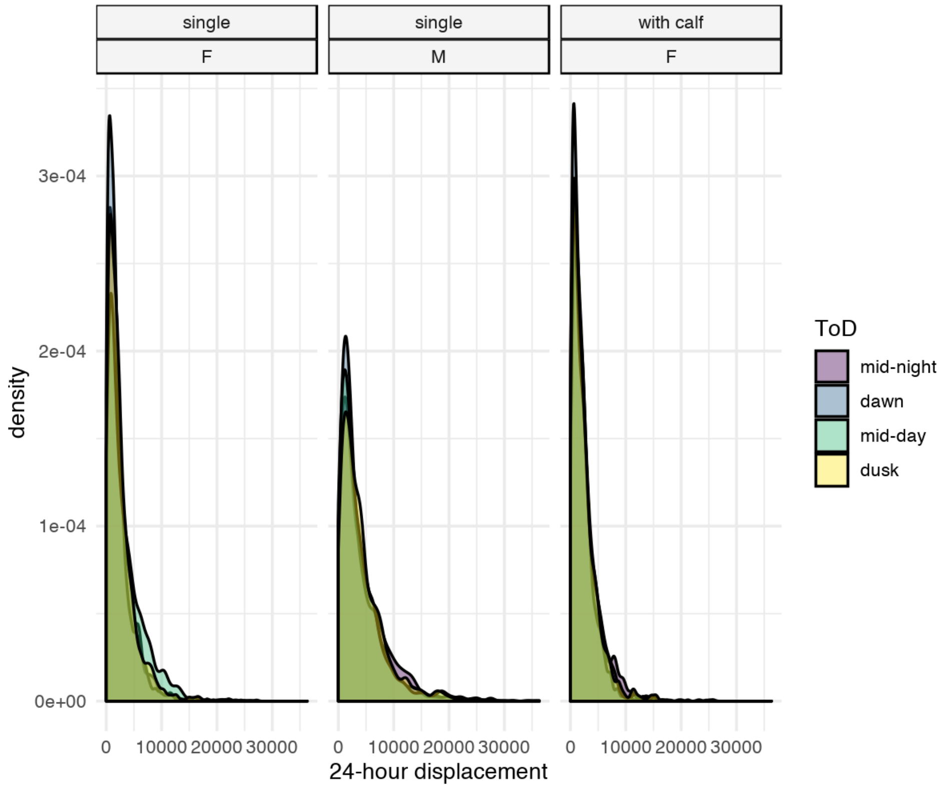

Figure 4 24-hour displacement across time of day and sex.

Appendix C: Biweekly Recursion: A null model

To fully interpret the relationship between recursion and NDVI, it is important to consider the underlying distribution of NDVI. In accordance with the methods described in the main text, meanNDVI was calculated for all grid cells created by ‘tlocoh‘ 16-day time use maps, including surrounding grid cells to which individuals did not visit within the 16 day interval. Visualizing the mean NDVI for all 1 km^2 grids, a prominently bimodal distribution is evident, echoed in the distribution of meanNDVI for those grid cells that were visited at least once (Fig. 5).

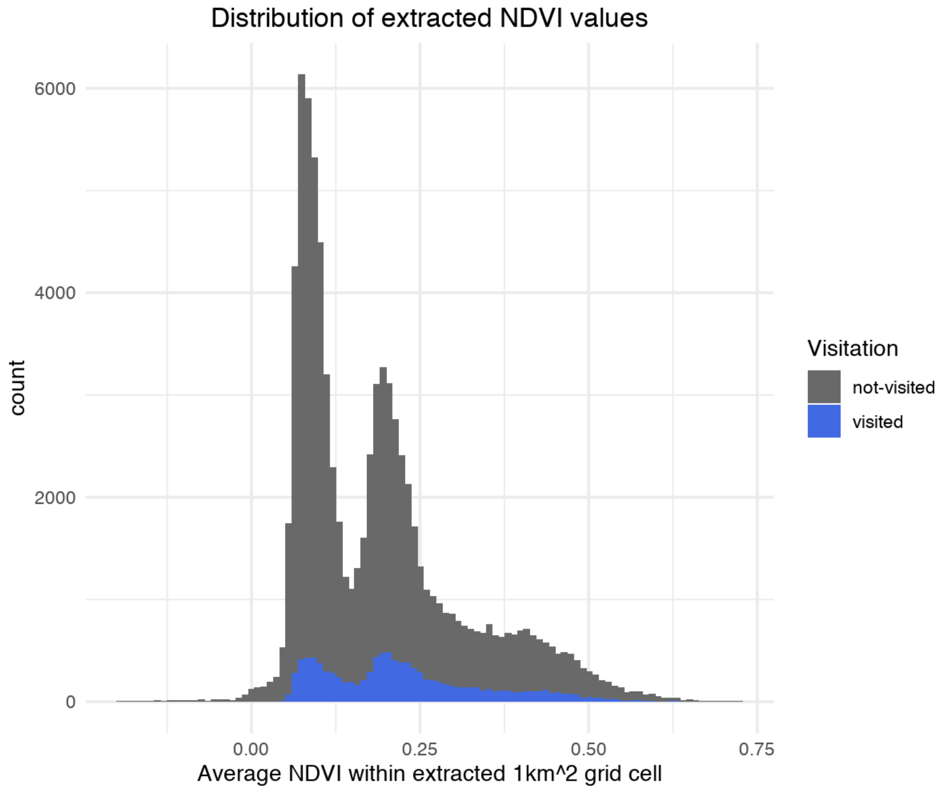

Figure 5 Distribution of mean NDVI from extracted cells.

To statistically evaluate if individuals were exhibiting a revisitation preference for areas of higher or intermediate NDVI more frequently than random, we compared our results to a null model shuffling (i.e. randomly sampling without replacement) meanNDVI values for each grid cell visited (but not necessarily revisited) by an individual during the course of their full trajectory. This provides an appropriate null model for the expected distribution of NDVI values if individuals were choos-

ing to revisit patches (within each given 16 day interval) at random, rather than exhibiting a preference explained by NDVI.

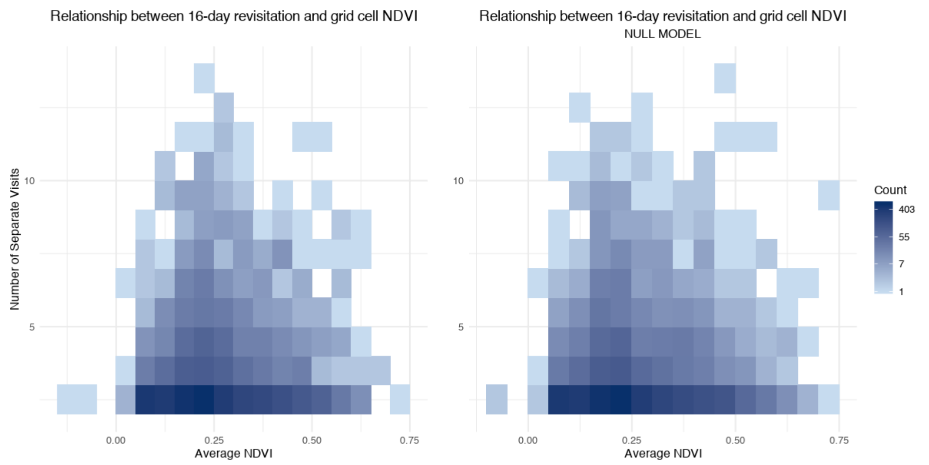

Figure 6 Side by side visualization of empirical and null revisitation patterns.

Comparing distribution of revisits, in the style of Figure 7 from the main text (Fig. 6), it is clear that the pattern of revisitation is quite similar across null and non-null models. To some extent this is expected given the coarse resolution of the data and that data are shuffled within (not across) individual trajectories. Even so, a narrower cone of high revisitation is visible in the empirical data as compared to the null (randomized) dataset.

We fit linear models to the null (shuffled) data and the empirical mean NDVI values for each cell (Eq. 1 & 2). Additionally we fit quadratic models to test our assertion they are selection for intermediate values of NDVI (Eq. 3 & 4; Table 2). The quadratic model fit best according to AIC (Table 3), fitting a hill like curve to the average NDVI values at revisited cells, an improved fit over even the quadratic null model (Fig. 7).

$$Revisitation = \beta_0 + \beta_1 x_{nullNDVI} \tag{1}$$

$$Revisitation = \beta_0 + \beta_1 x_{meanNDVI} \tag{2}$$

$$Revisitation = \beta_0 + \beta_1 x_{nullNDVI} + \beta_2 x^2_{nullNDVI} \tag{3}$$

$$Revisitation = \beta_0 + \beta_1 x_{meanNDVI} + \beta_2 x^2_{meanNDVI} \tag{4}$$

Table 2

Model	Resid. Df	Resid. Dev	Deviance
Model 1: nsv.43200 $\sim nullNDVI\_id$	10730	9286.7	
Model 2: nsv.43200 $\sim meanNDVI$	10730	9255.7	31.1
Model 3: nsv.43200 $\sim poly(nullNDVI\_id, 2)$	10729	9214.2	41.5
Model 4: nsv.43200 $\sim poly(meanNDVI, 2)$	10729	9101.2	112.9

Table 3

	df	AIC	dAIC
Model 1	2	34778.39	
Model 2	2	34747.33	-31.06
Model 3	3	34707.80	-39.53
Model 4	3	34594.86	-112.94

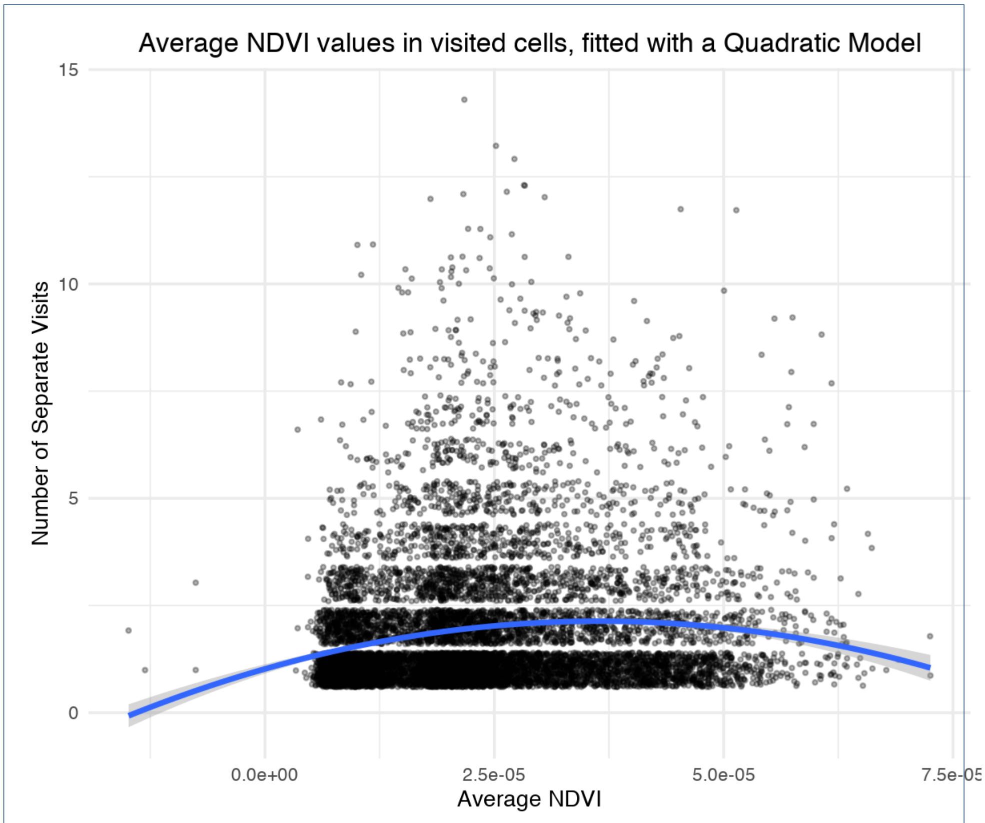

Figure 7 Quadratic fit to short-term revisitation to grid cells and local average NDVI

Appendix D: Home Range Analysis: Comparison of methods

To compare across available home range methods, we built utilization distributions using two methods, an autocorrelated kernel density estimator using the ‘ctmm‘ package and the k-LoCoH method using the ‘tlocoh‘ package. For both methods we extracted the area from the estimated 90% isopleths for comparison and regression with extracted NDVI.

Consistent with the main text, all continuous variables (area, and all NDVI variable: mean, min, max, median, sd) were normalized (i.e. scaled 0-1) and area was log-transformed before fitting models. Using a response variable of estimated home range area in square kilometers and running regressions on the 2 estimate sets independently (klocoh and akde estimates respectively), the inclusion of ‘id‘ as a random intercept variable significantly improved the fit of the models. Coefficient estimates indicating an inverse relationship between area and greenness (as expected) were stable and significant both models. Area estimates using akde were, on average, 10 times bigger than the k-LoCoH estimates.

Table 4 **k-LoCoH Regression results**

	Value	Std.Error	DF	t-value	p-value
(Intercept)	16.25	0.19	393	84.82	0.000
meanNDVI	-1.67	0.54	393	-3.07	0.002

Table 5 **Autocorrelated kernel density estimator (akde) Regression results**

	Value	Std.Error	DF	t-value	p-value
(Intercept)	18.18	0.23	447	78.86	0.00
meanNDVI	-1.52	0.72	447	-2.15	0.03

In the specific case of Etosha, where individuals’ home ranges commonly surround or abut actively avoided and unused areas (i.e. the Etosha pan), the k-LoCoH method was deemed most appropriate for its ability to identify home ranges respecting such strict boundaries.

Appendix E: Parameter Sensitivity

The buffer distance from watering holes chosen to analyse the frequency of fixes by time of day, was arbitrary but a sensitivity analysis demonstrates that the pattern found at a buffer of 250m holds across multiple buffer radii (Fig. 8).

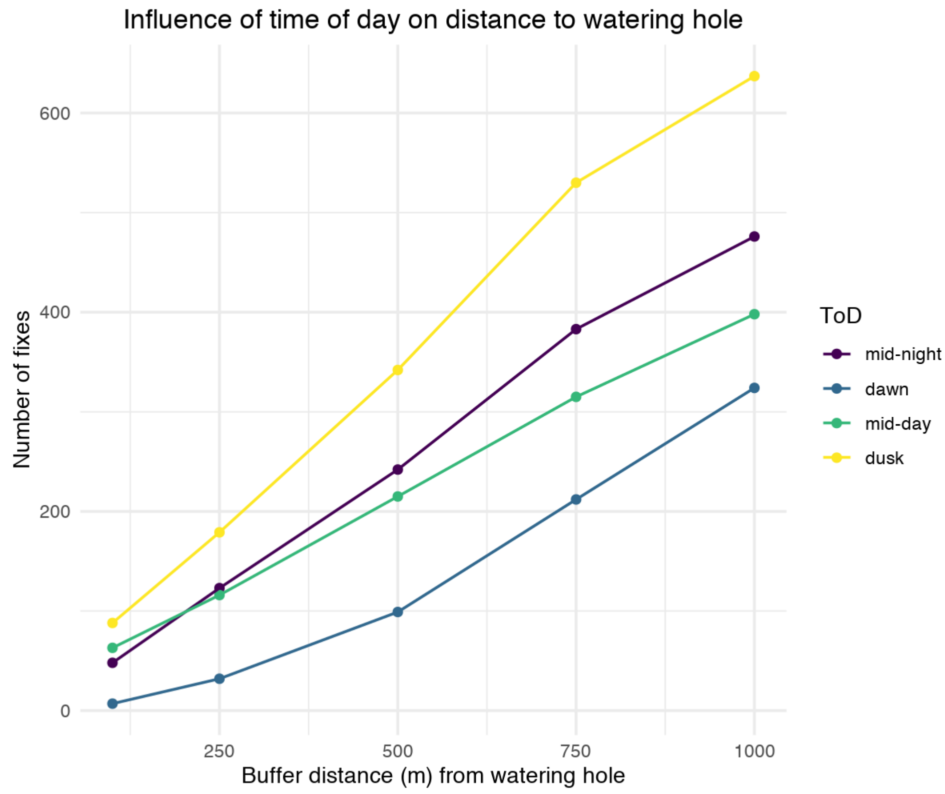

Figure 8 Relationship between Time of Day and distance from watering holes. Regardless of buffer distance, the pattern holds of dusk being the most common time of day to find rhinos near watering holes and dawn being the least.
